# Assistive AI in Lung Cancer Screening: A Retrospective Multinational Study in the United States and Japan

**DOI:** 10.1148/ryai.230079

**Published:** 2024-03-13

**Authors:** Atilla P. Kiraly, Corbin A. Cunningham, Ryan Najafi, Zaid Nabulsi, Jie Yang, Charles Lau, Joseph R. Ledsam, Wenxing Ye, Diego Ardila, Scott M. McKinney, Rory Pilgrim, Yun Liu, Hiroaki Saito, Yasuteru Shimamura, Mozziyar Etemadi, David Melnick, Sunny Jansen, Greg S. Corrado, Lily Peng, Daniel Tse, Shravya Shetty, Shruthi Prabhakara, David P. Nadich, Neeral Beladia, Krish Eswaran

**Affiliations:** From Google Health Research, 1600 Amphitheatre Pkwy, Mountain View, CA 94043 (A.P.K., C.A.C., R.N., Z.N., C.L., J.R.L., D.A., S.M.M., R.P., Y.L., S.J., G.S.C., L.P., D.T., S.S., S.P., K.E.); Waymo, Mountain View, Calif (J.Y., N.B.), David Geffen School of Medicine at UCLA, Los Angeles, Calif (C.L.); Google, Mountain View, Calif (W.Y.); Department of Gastroenterology, Sendai Kousei Hospital, Sendai, Japan (H.S.); MNES Inc, Hiroshima, Japan (Y.S.); Department of Telemedicine, Northwestern University Feinberg School of Medicine, Chicago, Ill (M.E., D.M.); and Center for Biological Imaging, New York University–Langone Medical Center, New York, NY (D.P.N.).

**Keywords:** Assistive Artificial Intelligence, Lung Cancer Screening, CT

## Abstract

**Purpose:**

To evaluate the impact of an artificial intelligence (AI) assistant for lung cancer screening on multinational clinical workflows.

**Materials and Methods:**

An AI assistant for lung cancer screening was evaluated on two retrospective randomized multireader multicase studies where 627 (141 cancer-positive cases) low-dose chest CT cases were each read twice (with and without AI assistance) by experienced thoracic radiologists (six U.S.-based or six Japan-based radiologists), resulting in a total of 7524 interpretations. Positive cases were defined as those within 2 years before a pathology-confirmed lung cancer diagnosis. Negative cases were defined as those without any subsequent cancer diagnosis for at least 2 years and were enriched for a spectrum of diverse nodules. The studies measured the readers’ level of suspicion (on a 0–100 scale), country-specific screening system scoring categories, and management recommendations. Evaluation metrics included the area under the receiver operating characteristic curve (AUC) for level of suspicion and sensitivity and specificity of recall recommendations.

**Results:**

With AI assistance, the radiologists’ AUC increased by 0.023 (0.70 to 0.72; *P* = .02) for the U.S. study and by 0.023 (0.93 to 0.96; *P* = .18) for the Japan study. Scoring system specificity for actionable findings increased 5.5% (57% to 63%; *P* < .001) for the U.S. study and 6.7% (23% to 30%; *P* < .001) for the Japan study. There was no evidence of a difference in corresponding sensitivity between unassisted and AI-assisted reads for the U.S. (67.3% to 67.5%; *P* = .88) and Japan (98% to 100%; *P* > .99) studies. Corresponding stand-alone AI AUC system performance was 0.75 (95% CI: 0.70, 0.81) and 0.88 (95% CI: 0.78, 0.97) for the U.S.- and Japan-based datasets, respectively.

**Conclusion:**

The concurrent AI interface improved lung cancer screening specificity in both U.S.- and Japan-based reader studies, meriting further study in additional international screening environments.

**Keywords:** Assistive Artificial Intelligence, Lung Cancer Screening, CT

*Supplemental material is available for this article.*

Published under a CC BY 4.0 license.

## Graphical abstract

**Figure:**
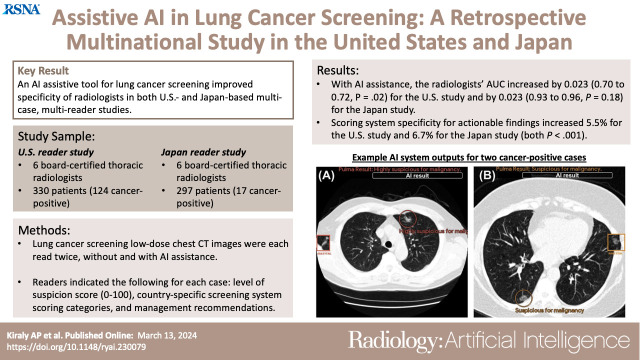

**Summary**

An artificial intelligence system and approach for surfacing artificial intelligence results to radiologists for lung cancer screening increased reader specificity in U.S.-based and Japan-based randomized retrospective reader studies.

**Key Points**

■ We developed and optimized an artificial intelligence (AI)–assistive workflow for lung cancer screening and tested it in the United States and Japan.
■ In two randomized retrospective reader studies (United States and Japan) involving challenging screening cases, experienced radiologists, and the institutional picture archiving and communication systems (PACS) viewers, an AI assistant improved specificity by 5%–7% with no significant drop in sensitivity.
■ Key findings applicable to other studies include effective communication of AI results to radiologists and an open-source software library offering wide PACS compatibility.

## Introduction

Lung cancer is the leading cause of cancer death globally and was responsible for 1.8 million deaths in 2020 (1). Five-year survival rates for lung cancer trail survival rates for other cancers, largely because of late diagnosis (2). Large randomized controlled trials have shown that lung cancer screening (LCS) programs applied to high-risk populations using low-dose chest CT (LDCT) can reduce lung cancer–specific mortality by at least 20% (3,4). To benefit a larger population, the United States Preventive Services Task Force has recently expanded screening eligibility by lowering requirements for both age and smoking history, increasing the eligible population by roughly 80% (3,5).

Although LCS availability has expanded worldwide over the last decade, patient enrollment has been modest, leading to concerns that high-risk populations are not fully realizing the potential benefits. Additionally, there are concerns regarding whether LCS performance in real-world clinical settings will mirror trial results conducted in controlled research environments. Real-world factors such as increasing radiologist workloads and variations in reader experience may also influence outcomes.

Artificial intelligence (AI)–based tools for nodule detection and characterization have demonstrated benefits for the clinical workflow, but barriers, including poor clinical integration, hinder widespread adoption (6). Lung cancer AI models have demonstrated performance on par with radiologists and even have shown superiority to existing guidelines (6–11). However, few studies have examined how such models can be incorporated into routine clinical workflows to assist radiologists. For nonscreening chest CT images, Dotson et al (12) demonstrated that AI recommendations for manually selected nodules can improve reader’s follow-up recommendations and increased comparative suspicion levels by 5.95%. Even fewer such studies have examined the impact of fully automatic AI models (that require no input such as nodule selection). LCS workflow integrations must also account for country-specific guidelines such as the American College of Radiology Lung CT Screening Reporting and Data System (ACR Lung-RADS) (13).

In this work, we evaluated the impact of a generalized AI-assistance system on LCS workflows in randomized retrospective reader studies under institution-specific workflow environments and equipment as well as country-specific guidelines scoring systems and management protocols. The cases that were selected were challenging in that the cancer cases were less suspicious and the negative cases were more suspicious based on past clinical history and imaging findings. Evaluations focused on multiple aspects, areas under the receiver operating characteristic curve (AUC) derived from reader’s level of suspicion (LoS) score for cancer and sensitivity and specificity based on guidelines and management. To understand generalizability, we investigated the impact of AI assistance on LCS for both U.S.- and Japan-based settings and patient populations.

## Materials and Methods

**Figure 1:**
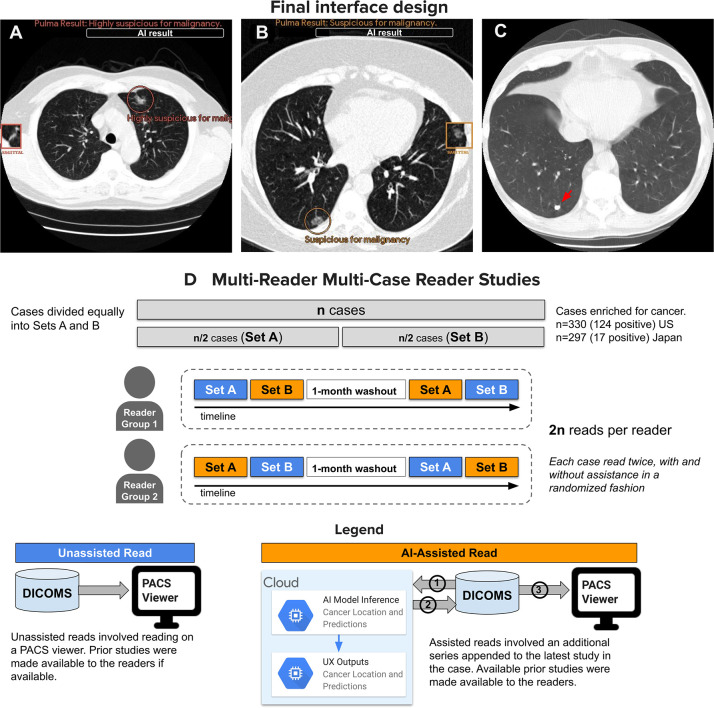
Overview of the final design and reader studies. **(A–C)** Artificial intelligence (AI) system outputs are designed to effectively communicate results to radiologists. Color-coded regions of interest (ROIs) and text-based suspicion categories are rendered over low-dose axial CT images. “Title slide” Digital Imaging and Communications in Medicine (DICOM) images (not shown) are placed prior to any results, allowing the reader to decide when to reveal the AI results. The overall case-level cancer suspicion category is rendered at the top of each section (above the “AI result” white box), while up to three localized ROIs are circled with the corresponding nodule-level suspicion text underneath. Nearby squares present the circled ROI sagittal view to better visualize three-dimensional morphology. All AI outputs are in DICOM format for easy integration into any picture archiving and communication system (PACS) viewer. No ROIs are presented for cases deemed negative. **(A, B)** Two cancer-positive cases (**[A]** 63-year-old male patient and **[B]** 63-year-old female patient) where the AI system assisted in increasing the radiologists’ suspicion. **(C)** A patient negative for cancer (66-year-old male patient) where the AI system decreased suspicion of a radiologist-identified solid nodule in the posterior right lower lobe (red arrow). **(D)** Two multireader multicase reader U.S.- and Japan-based studies were conducted on the selected cases with the same AI interface for both studies. A total of 14 experienced board-certified radiologists (12 effective; six per study) participated in the studies. Each case consisted of a selected study and any prior studies if available. AI outputs were derived from a single series in the current study. All cases were read twice by every reader, once with AI assistance and once without AI assistance (in a randomized order), with a 1-month washout period in between.

We conducted two multireader multicase reader studies comparing performance with and without AI assistance in the U.S.- and Japan-based reader and patient populations using a cloud-based AI system (Fig 1). See Appendix S2 for the AI system details. LDCT studies were collected from four sources: two sources from the United States, one source from Canada, and one source from Japan. All image data were stored in the Digital Imaging and Communications in Medicine (DICOM) format. Institutional review board approval was granted for each dataset in their respective locations, and all datasets were de-identified prior to transfer. The Advarra Institutional Review Board reviewed and granted a waiver for further review of these retrospective reader studies.

### Reader Studies

Six composite readers (see U.S. Study Details) participated in the U.S. and Japan study. Each reader interpreted half the cases without AI assistance and the other half with AI assistance. After a 1-month washout period, the readers then interpreted the same cases using the opposite method (Fig 1D), resulting in each reader reading each case twice. Prior to both studies, an introductory presentation was given to walk through each of the questions asked per case. The purpose behind each question was covered along with a calibration guide of how to report the 0 to 100 LoS score. The AI system background and interface was presented, and a small pilot study of five cases was run to ensure comprehension. For the Japan study, relevant materials were translated into Japanese. Readers were informed that the case set was cancer enriched but were not given an exact percentage.

For each evaluation, the readers provided the following: *(a)* an LoS value between 0 and 100 inclusive to indicate their suspicion of malignancy for the patient as a whole, *(b)* a score based on the respective country-specific scoring system (Lung-RADS v1.1 score for United States and Sendai score for Japan), and *(c)* a case management recommendation (readers could choose a recommendation different from those in the guidelines). All responses were evaluated against a single ground truth of cancer positivity within 2 years of imaging.

### U.S. Study Details

The U.S. study involved 330 patients (median age, 63 years [range, 49–82 years]; 191 male and 139 female patients) and a total of eight (six effective) U.S. board-certified thoracic radiologists (mean years of experience, 17 [range, 7–30 years]). Midway through the study, two readers became unavailable and were replaced by two new readers. Thus, an effective total of six radiologists participated in the study. All paired reads of the same case were conducted by the same reader. For the country-specific scoring system, radiologists applied Lung-RADS score (version 1.1) from the ACR guidelines (13). Readers used the eUnity web-based picture archiving and communication system (PACS) viewer (Mach7 Technologies) to read cases.

### Japan Study Details

Six Japan-based board-certified radiologists with experience in reading CT chest examinations (mean years of experience, 22.7 [range, 10–40 years]) and 297 patients (median age, 58 years [range, 31–83 years]; 217 male and 80 female patients) participated in the Japan reader study using the Sendai score system (14,15). The MNES LOOKREC PACS (16) was used for reading cases.

### Case Selection

Table 1 summarizes the datasets used in both studies, which involved datasets named DS_NLST, DS_US, and DS_JPN (see Appendix S2). The studies were enriched for clinically difficult cases, such as cancer cases that received less immediate follow-up recommendations and cancer-negative cases with suspicious nodules that resulted in more immediate follow-ups, where possible. Lower performance compared with a general population was more likely due to these more difficult cases. For positive cases, in both the U.S. (*n* = 124) and Japan (*n* = 17) studies, all available individuals with cancer were selected, choosing the earliest LDCT study that was within 2 years of the patient’s pathology-confirmed cancer diagnosis date. To ensure that these cases did not precede the lung cancer development itself, a board-certified radiologist with 19 years of experience (C.L.) verified that a nodule corresponding to the biopsied lung nodule was present in the selected study. Among the nodules found in positive cases in the U.S. study, 69 of 124 (56%) were solid, 15 of 124 (12%) were part-solid, 20 of 124 (15%) were nonsolid, and 29 of 124 (23%) were unclassified. Similarly, among the 17 nodules found in positive cases from the Japan study, six of 17 (35%) were solid, six of 17 (35%) were part-solid, and five of 17 (29%) were nonsolid. All available negative cases (*n* = 280) were included in the Japan reader study. For the U.S. study, negative cases comprised a subset of DS_NLST and DS_US negative cases. In DS_NLST, all cases with negative biopsies were first chosen (*n* = 54). Next, 150 cases were selected based on nodule size with 30 cases per category (no nodules, 2–6 mm, 6–8 mm, 8–15 mm, and 15 mm+). In DS_US, 27 presumed negative cases were selected with all cases with biopsies selected first followed by random negative cases. In each reader study, available prior studies were included for cancer cases. For negative cases, prior studies were selected randomly such that the proportion of cases with prior studies matched that of the cancer cases.

**Table 1:**
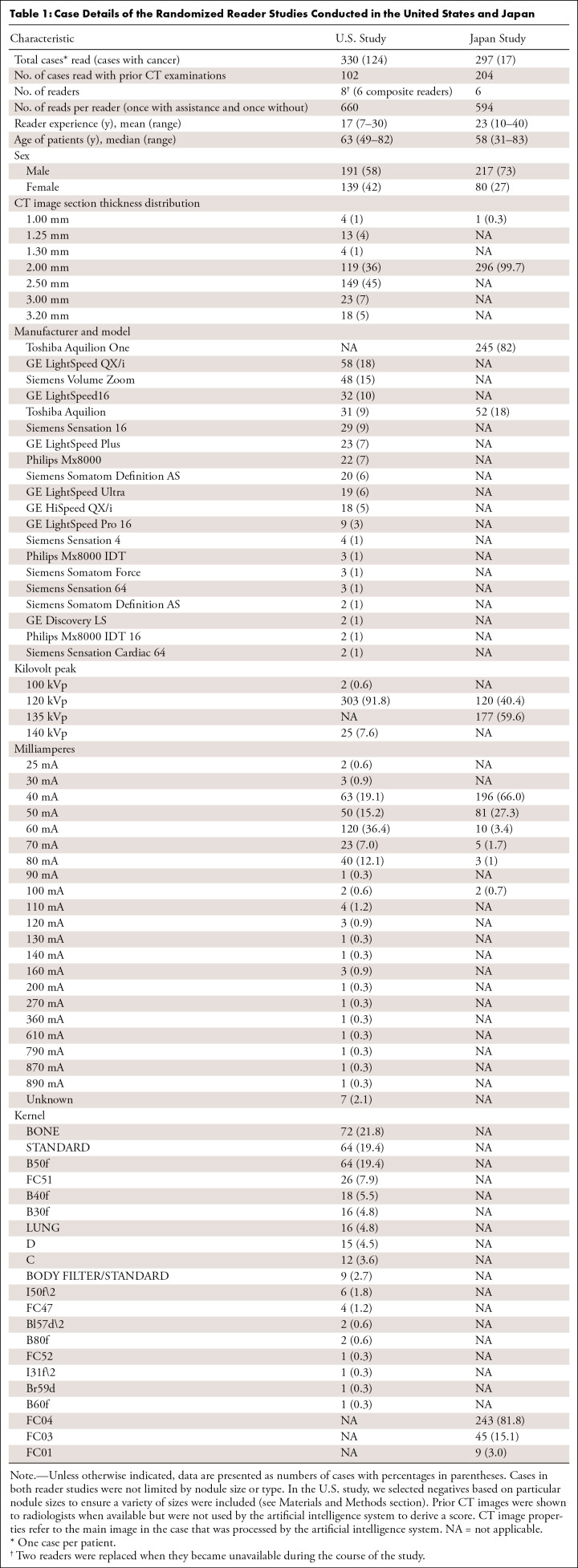
Case Details of the Randomized Reader Studies Conducted in the United States and Japan

### Statistical Analysis

The primary end point was the AUC derived from the LoS. The receiver operating characteristic curves for LoS were derived by sweeping all possible numerical thresholds to compute sensitivity and specificity. We used AUC because of its holistic measure of performance, capturing readers’ sensitivity across all possible specificities (17). Secondary end points included the radiologists’ sensitivity and specificity using the local scoring system and case management decision thresholds. For these secondary end points, sensitivity and specificity were computed on a binarized threshold based on urgency. For example, “follow-up CT in 6 months” was considered less urgent than “follow-up CT in 3 months,” which is in turn less urgent than “suspicious for malignancy.” Hence, a threshold of “less urgent versus 6 mo. follow-up+” involves counting more urgent management decisions as positive, resulting in higher sensitivity metrics for less urgent thresholds. Similarly, the same analysis was done for scoring system responses as well as the AI system responses. LoS AUC differences were compared using a one-sided two-treatment Obuchowski-Rockette-Hillis analysis (18) to evaluate for superiority and compute CI differences. A one-sided test was used because the only meaningful effect direction was if AI assistance improved performance. A *P* value of less than .05 indicated statistical significance for such AUC difference comparisons. *P* values for sensitivity and specificity comparisons were computed using a standard permutation test (19) with a sample size of 10 000 random data resamplings on the case level. More specifically, for each resampling, the complete assisted-unassisted reader responses were randomly swapped per case. A two-sided hypothesis test comparing the assisted-unassisted difference with the distribution of 10 000 assisted-unassisted differences across the resampled data was used to obtain an empirical *P* value. Significance for sensitivity and specificity pairings were evaluated individually based on the number of comparisons (four or five) in the set, hence a *P* value of less than .0125 or less than .01, respectively, was used to indicate statistical significance after a Bonferroni correction. CIs were computed using the nonparametric bootstrap method with a sample size of 1000 case-level resamplings for singular sensitivity, specificity, and AUC values as well as for sensitivity, specificity, and time differences. For each resampling, metrics were computed per reader and then averaged, or in the case of the AI system, taken directly. The percentile method was used to obtain the 95% CIs. Interreader agreement was assessed using two-way mixed, absolute, average intraclass correlation coefficient with a fixed set of readers (20). Scikit-learn 1.0.2, the software listed in the next section, and custom Python code running under Python 3.11 (Python Software Foundation) were used to perform analysis.

### Data and Code Availability

This study used one publicly available dataset, NLST *(https://biometry.nci.nih.gov/cdas/learn/nlst/images/)*. The datasets from Northwestern Medicine, the Canadian hospital system, and Japan were used either under license or a research agreement for the current study and are not publicly available. Interested researchers should contact *mozzi@northwestern.edu*, *radiologyservices@realtimemedical.com*, and *h.saito0515@sendai-kousei-hospital.jp* for access, respectively.

Python implementations of the statistical approaches used have been made available at *https://github.com/Google-Health/google-health/tree/master/analysis*. DICOM processing for AI models and a general library and framework to create PACS-accessible DICOMs from AI models for reader studies are available at *https://github.com/Google-Health/google-health/tree/master/ct_dicom*.

Finally, the AI models used for this study are licensed to DeepHealth (RadNet) and Apollo Hospitals. Interested researchers may contact *sorensen@deephealth.com* or *info@apolloradiology.ai*, respectively.

## Results

### Reader Studies: Radiologist Performance

**Figure 2:**
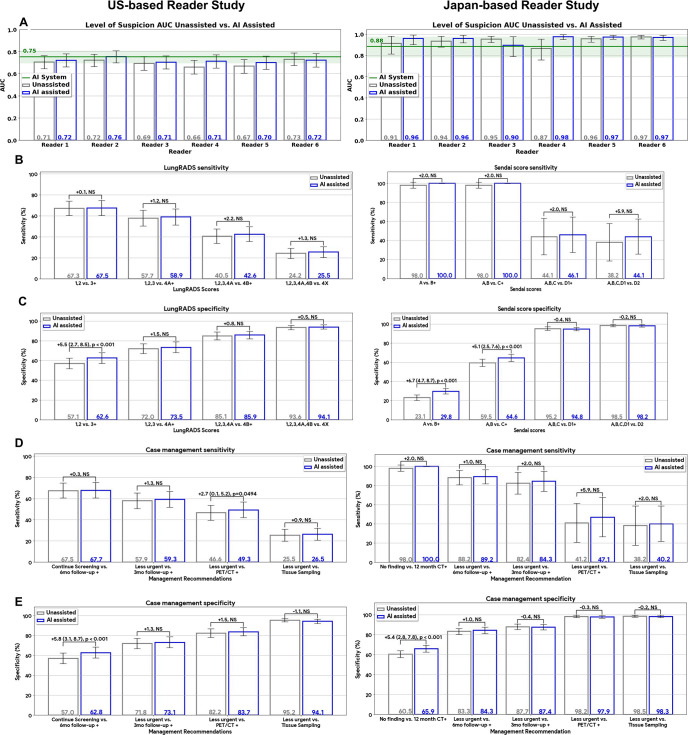
Reader performance with and without artificial intelligence (AI) assistance for U.S.- (left) and Japan-based (right) studies. Readers provided their level of suspicion (LoS) for lung cancer, country-specific scoring system scores (Lung Imaging Reporting and Data System [Lung-RADS] v1.1 for the U.S. study or Sendai score for the Japan study), and case management recommendations. Gray and blue bars represent metrics without and with AI assistance, respectively. **(A)** Area under the receiver operating characteristic curve (AUC) values computed from the LoS scores demonstrated an average improvement of 0.023 with AI assistance in both studies (*P* = .02; *P* = .18). In each study, five of six individual reader AUC scores were greater with AI assistance. The stand-alone AI system performance is shown as a green line. **(B, C)** Country-specific scoring system metrics for **(B)** sensitivity and **(C)** specificity (Lung-RADS left, Sendai score right). Though not significant (NS), average sensitivity was greater across all categories under AI assistance in both studies. For the U.S.-based study, the specificity of Lung-RADS 3+ increased 5.5% with AI assistance (*P* < .001) with no evidence of a difference in specificity between groups for all other categories. For the Japan-based study, specificity increased 6.7% (*P* < .001) and 5.1% (*P* < .001) for Sendai scores B+ and C+, respectively. There was no evidence of a difference in specificity between groups for the other two score groupings. **(D, E)** Case management–based metrics for **(D)** sensitivity and **(E)** specificity. Plots show metrics at less urgent versus more urgent junctions, as listed in the horizontal axes. With AI assistance, specificity increased 5.8% (*P* < .001) and 5.4% (*P* < .001) in the U.S.- and Japan-based studies for any actionable recommendations, respectively.

Radiologists’ AUC values were computed using their numeric LoS scores, and two sets of sensitivity and specificity metrics were derived from the country-specific scoring system and case management responses (Fig 2). In both the U.S. and Japan studies, relative to unassisted reads, AI assistance increased average reader sensitivity on all categories in the scoring systems and case management options (albeit not statistically significant), with statistically significant specificity increases for grouped categories involving any suspicious findings (*P* < .001).

### U.S. Study

**Figure 3:**
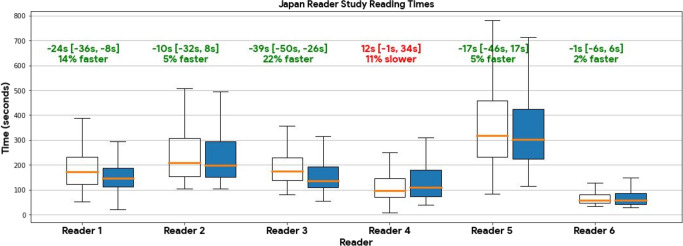
Interpretation times with and without artificial intelligence (AI) assistance for each reader in the Japan-based reader study. Median per-case reading times and differences in seconds per reader with and without AI assistance in the Japan-based reader study are plotted in orange on boxplots of unassisted (clear) and assisted (blue) reading times. First and third quartiles are the lower and upper extent of the boxes, respectively. The whiskers represent the minimum and maximum values. Outliers are not shown. Times were based on the timestamp of submission subtracted from the previous submission with filtering for times over 20 minutes to account for breaks in reading or starting tasks. The filtering was applied per reader at the patient level to both reading tasks. Due to technical issues in the timing implementation, times for the U.S.-reader study were unavailable. The average reader median reading time was 14 seconds (95% CI: 5, 20) less using AI assistance which amounts to an 8% faster reading time with AI assistance.

The U.S. reader study (left panels of Fig 3, Tables 2 and 3) demonstrated an LoS AUC improvement of 0.023 (0.70 to 0.72 [95% CI: 0.01, 0.045]; *P* = .02) with the averaged assisted reads achieving improved sensitivity and specificity across all Lung-RADS scores over the unassisted reads. For comparison, the underlying AI system’s stand-alone AUC was greater than the LoS AUC values of every reader for both assisted and unassisted reads except for one reader achieving a slightly higher AUC than the AI system when assisted. At the Lung-RADS 3+ threshold (ie, a positive screen), AI assistance increased specificity 5.5% (57% to 63% [95% CI: 2.7, 8.5]; *P* < .001). Similar results were obtained in readers’ case management decisions with a 5.8% (57% to 63% [95% CI: 3.1, 8.7]; *P* < .001) specificity increase for a 6-month follow-up CT examination or more urgent recommendation (ie, any case management recommendation resulting in more frequent imaging or procedures). Sensitivity and specificity scores were higher with AI assistance for all case management categories except for a nonsignificant drop in specificity in biopsy recommendations by −1.1% (95% to 94% [95% CI: −2.6, 0.2]; *P* = .1208). Figure S4 shows results on the NLST subset in the U.S. study reweighted to the NLST patient population, demonstrating the specificity remained increased 3.4% (83% to 86% [95% CI: 0.6, 6.3]; *P* = .011) for Lung-RADS 3+. The corresponding sensitivity and specificity of the AI system outputs are listed in Table 3.

**Table 2:**
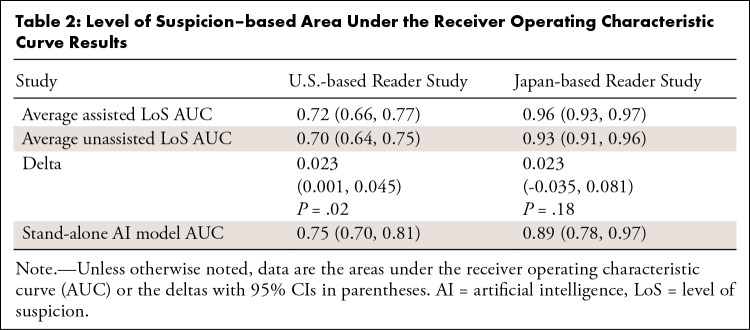
Level of Suspicion–based Area Under the Receiver Operating Characteristic Curve Results

**Table 3:**
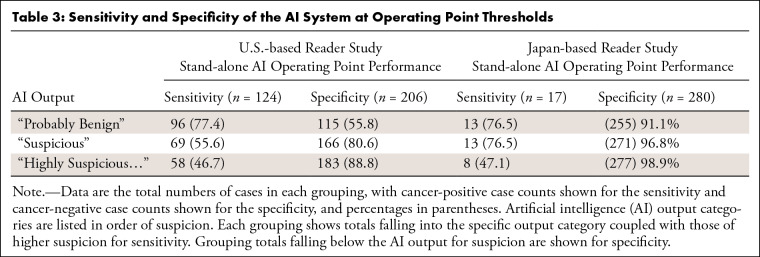
Sensitivity and Specificity of the AI System at Operating Point Thresholds

### Japan Study

The Japan reader study (right panels of Fig 3) also demonstrated an AI-assisted LoS AUC improvement of 0.023 (0.93 to 0.96 [95% CI; -3.5, 8.1]; *P* = .1789), though this did not reach statistical significance due to the smaller sample size. Statistically significant improvements were observed for specificity in categorical responses. Specificity increased 6.7% (23% to 30% [95% CI: 4.7, 8.7]; *P* < .001) for positive findings (ie, B or greater Sendai score; see Materials and Methods section) and 5.1% (60% to 65% [95% CI: 2.5, 7.6]; *P* < .001) for Sendai categories C and greater. Similar to the U.S. reader study, increases in sensitivity across all scores were observed. For actionable case management decisions (ie, any follow-up recommendation), an increased average reader specificity of 5.4% (61% to 66% [95% CI: 2.8, 7.8]; *P* < .001) was achieved. The model provided assistive benefits to readers despite its own stand-alone performance (AUC, 0.88; 95% CI: 0.78, 0.97) being below the LoS-based AUC of most readers (AUC, 0.93; 95% CI: 0.91, 0.96). This is likely partially due to the sensitivity (47.1%) and specificity (98.9%) of the AI system’s “Highly Suspicious” category being greater than the average unassisted reader’s most suspicious category response (38.2% and 98.5%) coupled with the surfaced locations allowing the radiologists to better selectively integrate the AI system results.

### Reader Studies: Additional Analyses

**Figure 4:**
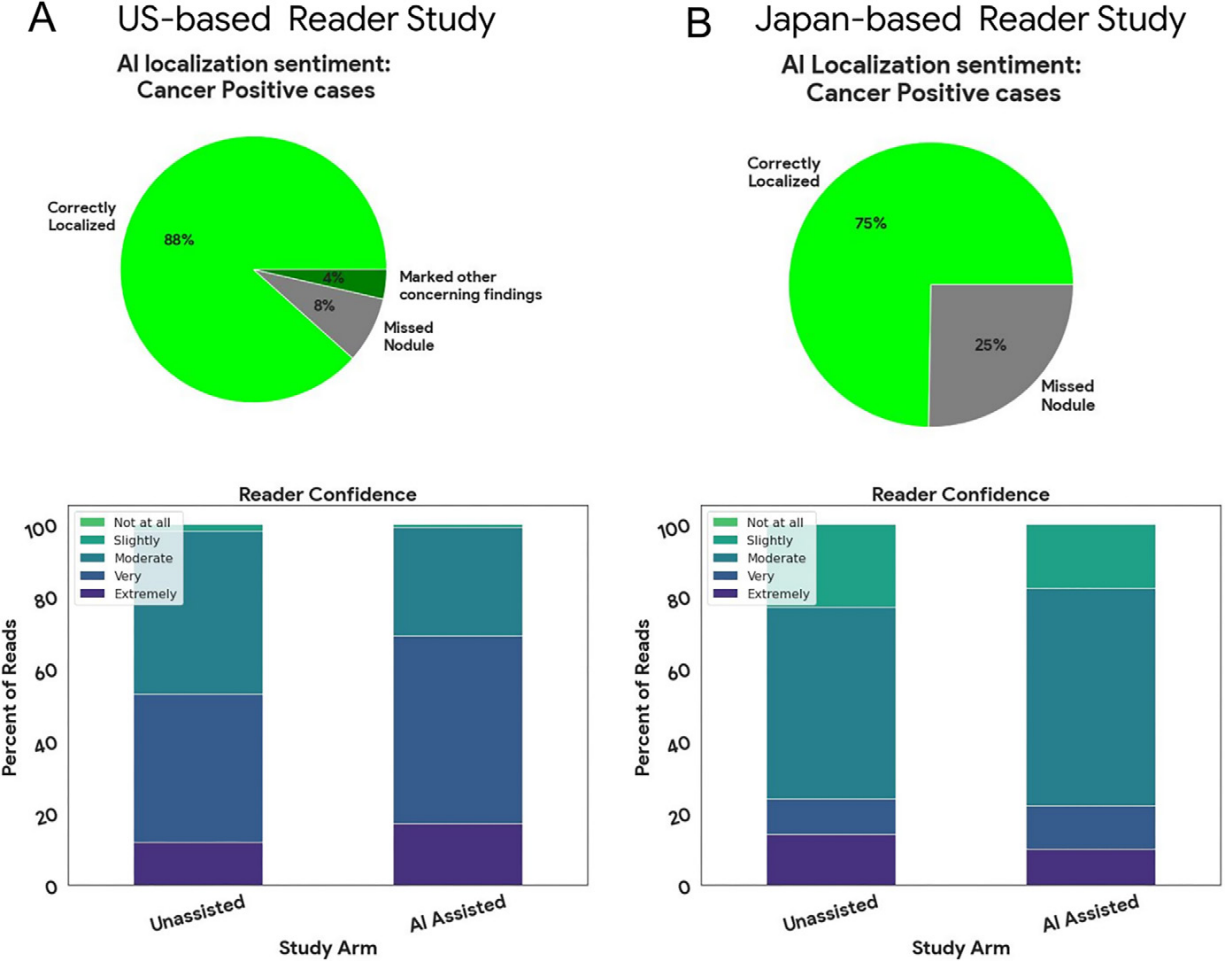
Reader sentiment on artificial intelligence (AI)–system nodule localization for cancer-positive cases (top) and reader confidence for all cases (bottom). Readers were asked multiple-choice questions about their thoughts on the AI localization (AI-assisted cases only) and their decision confidence on a per-case basis in the **(A)** U.S.-reader study and **(B)** Japan-reader study. Sentiment on AI nodule localization is shown for ground truth cancer-positive cases. Overall, the sentiment was that the system localized the most concerning nodule in 88% of the cases in the U.S.-reader study and 75% of the cases in the Japan-reader study. Confidence at moderate or higher levels increased with AI assistance in both studies, with a greater boost to U.S.-based readers.

**Figure 5:**
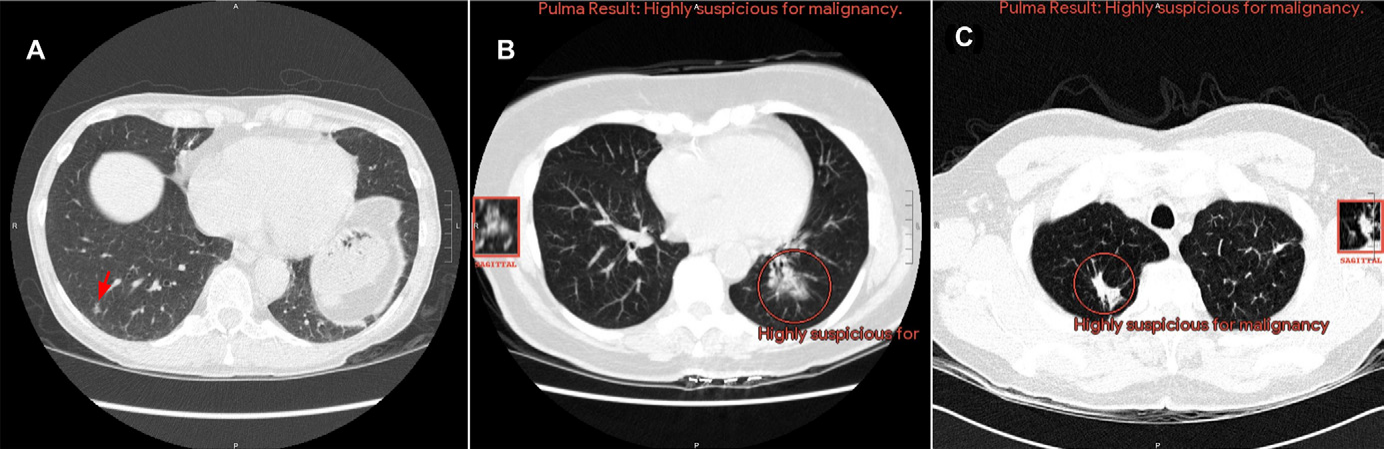
Illustrative examples of axial low-dose chest CT images with a missed lesion and false positives by the artificial intelligence system. **(A)**. A 69-year-old female patient with a missed malignant 7-mm subsolid nodule with irregular margins in the right lower lobe. **(B)** False positive in a 69-year-old female patient. CT image shows a nonmalignant part-solid heterogeneous opacity in central left lower lobe with features mimicking adenocarcinoma. **(C)**. False positive in a 66-year-old male patient. CT image shows a lung infection in the setting of severe emphysema presenting as a solid nodular opacity with irregular margins in the right upper lobe.

Reading times were available for the Japan reader study and show that assisted readers were 14 seconds faster per case on average (95% CI: 5, 20). This was also the case with five of six readers (Fig 3). Reader confidence also increased with AI assistance (Fig 4). In terms of AI-assisted localization, readers reported the AI system marked the most concerning nodule in 89% of the cancer cases in the U.S. study and 75% of the cancer cases in the Japan study (Fig 4). In the remaining cases, the readers found that the system missed the most concerning nodule. Although, for 4% of the U.S. cases, other concerning findings were marked. Increased reader agreement with AI assistance was noted across all response categories, as measured by higher intraclass correlation coefficient values compared with no AI assistance, though significance was not formally assessed (Table 4). In one case that readers marked suspicious while the AI system marked negative, smaller subsolid nodules were identified. These nodules were negative within our 2-year ground truth window (no cancer diagnosis within at least 2 years of negative follow-ups). Upon further radiologist review, they appeared to be cases of minimally invasive adenocarcinoma that were being managed by watchful waiting. Figure 5 shows examples where the AI assistant missed lesions or produced false positives.

**Table 4:**
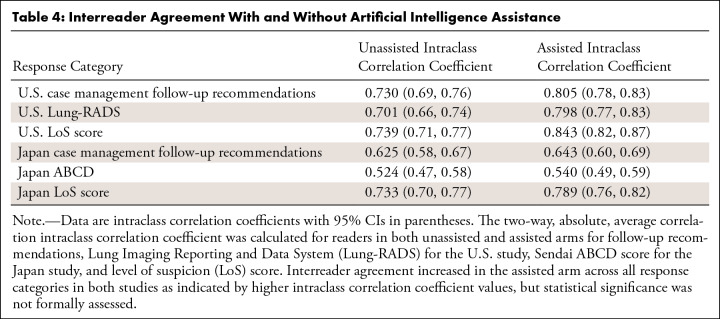
Interreader Agreement With and Without Artificial Intelligence Assistance

## Discussion

In this work, we evaluated an automatic AI-based LCS assistant with local PACS viewer integration. The key interface learnings may help guide designs of similar computer-aided diagnosis (CADx) systems. As LCS practices vary across institutions and countries with respect to the underlying screening and scoring protocols and management strategies, we opted for a more general approach presenting less prescriptive risk buckets for broader applicability. The resultant system demonstrated positive results and is worth exploring in further studies and environments. Specifically, these reader studies involving experienced radiologists reading difficult (ie, earlier cancer and negative suspicious nodules) lung cancer cases on LDCT images demonstrated that AI-assisted readers had increased specificity (*P* < .001) for metrics involving the threshold between a negative screen and any actionable findings under both scoring system and case management metrics in the U.S.- and Japan-based reader studies. Country-specific scoring system specificity across an actionable score showed absolute increases of 5.5% and 6.7% in the U.S.- and Japan-based reader studies, respectively. Specificity for actionable case management recommendations increased by absolute values of 5.8% and 5.4% for each study. LoS-based AUC increased by an identical effect size across both studies (2.3%), though statistical significance was reached only for the U.S. study (with more positive cases). The median reading time decreased by 14 seconds in the Japan-based study.

Different patient populations influence system performance, and unlike the U.S. study, the AI system obtained a lower AUC value versus the average unassisted reader (0.88 vs 0.93) in the Japan study. On stand-alone performance, the AI system had a lower AUC value for the Japan dataset (0.028 lower than DS_NLST). There are several factors that can account for the performance differences related to the different patient population and protocols. The underlying model was trained only on data from North America, which may have impacted generalizability. Specific reconstruction kernels and imaging devices in the Japan dataset were never seen during training. More importantly, low-grade adenocarcinomas with a predisposition to appear as slow-growing subsolid lung nodules have been observed to occur more frequently in nonsmokers and Asian female individuals (21). The Japan dataset contained a higher percentage of such part-solid nodules. Eligibility requirements were also broader in the Japan dataset and included younger individuals. This dataset also contained cancerous nodules with lower AI suspicion resulting in an early drop in sensitivity and overall AUC by the system.

Interestingly, the lower AUC value of the AI system relative to the average unassisted reader in the Japan study did not hinder the system in providing measurable benefits over unassisted interpretation. The AI system was more sensitive than readers in the more severe category, while less so in the “Probably Benign” category. Radiologists’ experience with the patient population and system training may have played a central role in this result, as readers were informed that the AI result is not always correct and factored the system result appropriately on a case-by-case basis. Japan study readers were asked for their sentiment on per-case AI usefulness (See Appendix S3). Under subgroup analysis of the cancer-positive cases, where the AI system had lower versus higher performance, readers found the AI was at least somewhat useful in 97% of cases in the higher performance subgroup and only 38% in the lower performance subgroup. Surfacing regions of interest may have contributed to readers being able to selectively factor in AI results. In other words, the Japan study demonstrated positive AI benefits via selective usage of the AI results by the readers. Similar conclusions were found in a previous study of chest radiographs (22). Further study using study designs including “sham AI” (ie, deliberately inaccurate AI) (22,23) applied to LCS may help shed light on how AI inaccuracies may influence user trust, though there are challenging and nuanced study design considerations such as whether to inform study readers of the sham nature of some AI outputs.

AI systems can potentially ameliorate some bottlenecks if they can increase productivity without compromising quality or disrupting workflows. Given the potential global impact, it is important that such a system could be generalized to different patient populations with varied baseline lung cancer risk profiles. Previous studies investigating AI assistance for lung cancer at chest CT focused on either nodule detection or nodule characterization based on manually selected nodules without detailing human interface aspects in the system design. To the best of our knowledge, no previous study was conducted internationally with different patient populations, overall cancer risk, and screening guidelines on a fully automatic system using more difficult cancer cases. Our study focused on the United States and Japan, between which several factors varied, including protocols, strategies, and patient populations.

While our AI system shows potential to impact LCS worldwide, the studies performed and the system both have limitations to consider. As in other recent studies, although realistic environments were used, the studies were retrospective on an enriched dataset. Similarly, this study did not include the full breadth of potential data that a radiologist may access in the electronic health record, such as smoking history. Any impact of having such variables available will need to be evaluated in future work. As our analysis principally focused on the performance of experienced radiologists on challenging cases, the impact of AI assistance on less experienced radiologists also needs to be examined in follow-up work. As positive cancer cases are more difficult to obtain, a total of 50 patients outside of NLST that were positive for cancer were used for evaluation. These cases were from different patient populations and involved selecting the earliest study within 2 years prior to biopsy. Subject to study feasibility, future studies could improve upon these limitations of retrospective enriched studies.

Several important confounding factors also bear mentioning. Although LCS is performed on asymptomatic individuals, actionable nononcologic disease may be present. For example, lung infection and lung cancer can mimic each other, confounding AI systems, cancer labeling, and radiologists (24). Cancer type was also a potential confounder. Minimally invasive lung adenocarcinomas often demonstrate a more indolent course than invasive lung malignancies and are often managed via successive rounds of follow-up LDCT. Consequently, such cases may not come to biopsy within the first 2 years after they are first recognized. This can affect labeling of a dataset since true (albeit low-grade) lung cancer cases of this type may be “truthed” as negative, impacting an AI model’s ability to accurately characterize subsolid lung nodules as lung cancer. As validation sets would face a similar situation, the ability to quantify the overall impact can be challenging. Nonetheless, it is reasonable to suspect that this (and other) AI systems designed to assess lung cancer risk may be less sensitive in identifying such cancers and less accurate in characterizing subsolid lung nodules. However, if the true end point of LCS is arguably decreasing lung cancer–specific mortality and maximizing quality-adjusted life years, the impact of minimally invasive lung cancers in both training and validation datasets may be limited.

In summary, LCS has been shown to be effective in mortality reduction in high-risk populations, but concerns remain around specificity and capacity, especially when the number of U.S. screening participants may dramatically increase (25). In this work, we evaluated an AI system that integrates into existing clinical reading workflows. The system demonstrated effectiveness in retrospective reader studies across two countries, PACS systems, and patient populations, as unnecessary follow-ups were reduced in interpreting challenging LCS cases with AI assistance. Incorporating such a system may reduce overaggressive use of follow-up imaging, avoidable lung biopsies, and burden on health care systems. Further studies using less experienced radiologists would give a better understanding of the overall impact. Surfacing AI outputs for radiologists is a complex combination of interaction, workflow efficiency, and efficacy. Reader training, design, and evaluations play an important role in evaluating AI systems (17). The generalized approaches and discussion presented can have broad applicability to a wide range of AI approaches to lung cancer and beyond.
